# Paramagnetic active sites boosted hydrogenation of *p*-nitroaniline over _pr_CeO_2_ supported Pt catalysts

**DOI:** 10.1039/d6ra00621c

**Published:** 2026-03-16

**Authors:** Hang Yu, Yuqing Li, Xiaoyang Yu, Jie Gao

**Affiliations:** a Department of Chemical Engineering, Sichuan University Chengdu 610065 China j.gao@scu.edu.cn

## Abstract

Developing efficient catalysts for *p*-nitroaniline hydrogenation under green conditions is pivotal for sustainable chemical synthesis. Herein, we report a Pt catalyst supported on a self-made _pr_CeO_2_ support that achieves >99% yield in the hydrogenation of *p*-nitroaniline to *p*-phenylenediamine under exceptionally green conditions (35 °C, 5 mg catalysts, 69 mg substrate, H_2_ balloon, 10 h, 2 mL methanol solvent). The superior performance is linked to paramagnetic active sites at the Pt–_pr_CeO_2_ interface, generated at an optimal reduction temperature of 400 °C, and enhanced hydrogen spillover *via* their unpaired electrons. After reusing it 20 times, it still retains 50% of its original performance. This work introduces paramagnetic active sites as a novel strategy for catalysis chemistry and synthesis.

## Abstract 2

The paramagnetic active sites were created at the Pt–_pr_CeO_2_ interface and generated at an optimal reduction temperature of 400 °C, and enhanced hydrogen spillover *via* their unpaired electrons.

## Graphical abstract

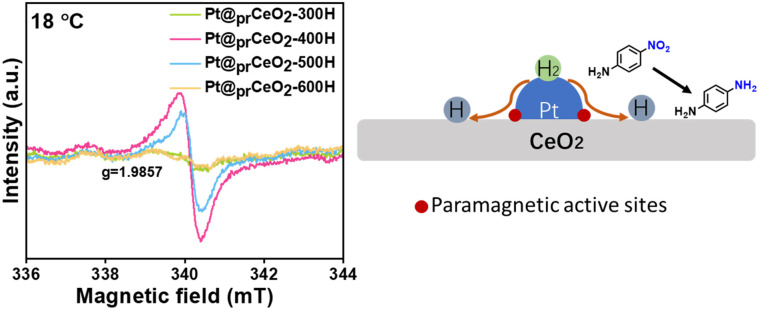

## Introduction

*p*-Phenylenediamine (PPD) is a crucial chemical intermediate with indispensable applications in the production of aramid fibers, high-performance dyes, rubber antioxidants, and epoxy curing agents. Its industrial synthesis typically relies on the catalytic hydrogenation of *p*-nitroaniline (*p*-NA).^1^ However, this reaction presents a significant scientific challenge: the strong electron-donating amine group (–NH_2_) in *p*-NA, through its resonance effect, substantially reduces the electron affinity of the para-nitro group (–NO_2_), rendering both the thermodynamic and kinetic activation and reduction of the nitro group particularly difficult.^2^ Numerous catalysts based on Pd, Pt, Rh, Ni, Co and Fe have focused on the hydrogenation of single nitro compounds. However, a persistent challenge is the high catalyst-to-substrate mass ratio, typically exceeding 30% for catalysts with a metal loading below 1 wt%. This limitation results in extended reaction times and the need for harsh conditions, such as temperatures above 50 °C and hydrogen pressures greater than 1 MPa. Consequently, developing highly efficient catalysts to achieve the selective hydrogenation of *p*-NA under green conditions (near ambient temperature and pressure) is of great scientific and industrial importance for reducing energy consumption, simplifying processes, and enhancing operational safety. This represents a promising yet challenging research direction.

In recent years, the electron spin state of catalytic active sites, particularly paramagnetism, has demonstrated unique potential and remarkable efficacy in regulating the performance of heterogeneous catalysis.^3–5^ Moving beyond the traditional focus solely on geometric and electronic structures, the paramagnetism of an active site (*i.e.*, the presence of unpaired electrons) offers novel pathways for the adsorption, activation, and conversion of reactant molecules.^6–8^ The mechanisms by which paramagnetism promotes catalysis primarily stem from the following aspects: (1) spin-dependent adsorption and activation: unpaired electrons at paramagnetic sites can engage in specific, strong interactions with reactant molecules possessing antiparallel spins (such as O_2_, NO_2_, or molecules that can generate radical intermediates *via* electron transfer) through spin-exchange interactions, effectively lowering the activation energy barrier. (2) Facilitation of electron transfer: paramagnetic sites are often associated with variable local electronic structures and can act as efficient electron “relay stations,” accelerating interfacial charge transfer, which is crucial for hydrogenation or reduction reactions involving multiple electron steps. (3) Modulation of reaction pathways: the spin state can influence the stability of key intermediates, thereby steering the reaction along a more favorable pathway, suppressing side reactions, and enhancing selectivity for the target product. Therefore, the deliberate construction and utilization of paramagnetic active sites opens a new dimension for designing next-generation high-performance catalysts. Cerium oxide, renowned for its exceptional oxygen storage/release capacity, flexible Ce^3+^/Ce^4+^ redox couple, and propensity to form oxygen vacancies, has emerged as a vital support for metal-loaded catalysts.^9–11^ It can create unique interfacial active sites through strong metal-support interactions (SMSI) with metal components.^12–14^ The Ce^3+^ ion, with its 4f^1^ electron configuration, is paramagnetic and is often considered a potential paramagnetic active site. However, Li and co-authors demonstrated that the paramagnetism origin of Ce based materials is not confined to Ce^3+^, indeed it is the oxygen vacancy promote the oxidation of Ni^2+^ to Ni^3+^, which is the true paramagnetic active sites.^15^

This study designed and prepared Pt catalysts supported on self-made _pr_CeO_2_ for the green hydrogenation of *p*-NA. Through systematic material characterization, we discovered and confirmed the existence of paramagnetic active sites within the system that are not sourced from Ce^3+^ ions. This finding challenges the conventional attribution of paramagnetism in ceria-based supports solely to Ce^3+^ species, suggesting the potential formation of novel paramagnetic sites induced by the complex interplay at the Pt–_pr_CeO_2_ interface. We investigate the nature and formation mechanism of this paramagnetic active site and elucidate its key role in significantly enhancing the activity and selectivity of the *p*-NA hydrogenation reaction. The study not only provides a high-performance catalyst for the efficient and mild hydrogenation of *p*-NA but also deepens the understanding of the role of paramagnetic sites in faciliating hydrogen spillover, offering new insights for the rational design of advanced catalytic materials used in green synthesis.

## Results and discussion

### Screening of catalysts

The selective hydrogenation of *p*-nitroaniline (PNA) to *p*-phenylenediamine (PPD) was chosen as a model reaction to evaluate the performance of various Pt-based catalysts under mild conditions (H_2_ balloon, 35 °C, 10 h). As summarized in Table 1, the catalyst support plays a decisive role. Pt nanoparticles supported on ZnO or Fe_2_O_3_ (Table 1, entries 1–2) showed negligible activity (<1% conversion), while Pt@Al_2_O_3_-400H (Table 1, entry 3) afforded a moderate yield of 19%. Strikingly, Pt supported on _pr_CeO_2_ (Pt@_pr_CeO_2_-400H) achieved quantitative conversion and yield (>99%, Table 1, entry 4), highlighting the superior efficacy of the _pr_CeO_2_ support. Screening of solvents using the optimal Pt@p-CeO_2_-400H catalyst revealed that protic solvents generally outperform aprotic ones (Table 1, entries 4–8). Methanol, offering good hydrogen solubility and potential H-bonding interactions, gave the best result (Table 1, entry 4). Isopropanol, another protic solvent, also showed high activity (90% yield, Table 1, entry 6), though slightly lower, possibly due to its larger steric bulk. In contrast, aprotic solvents like tetrahydrofuran (THF, 52% yield, Table 1, entry 7) and acetonitrile (MeCN, 18% yield, Table 1, entry 8) led to significantly reduced performance. This trend may be linked to their weaker ability to stabilize polar transition states or participate in proton-transfer steps. Notably, the reaction was completely suppressed in water (<1% yield, Table 1, entry 5), likely due to competitive adsorption of water on active sites, inhibition of reactant diffusion, or unfavorable thermodynamic equilibrium in the aqueous phase.

**Table 1:** The selective hydrogenation of *p*-nitroaniline over different catalysts^a^

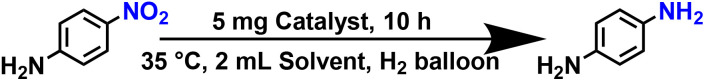				
Entry	Catalyst	Solvent	Conversion, %	Yield, %
1	Pt@ZnO-400H	MeOH	<1	<1
2	Pt@Fe_2_O_3_-400H	MeOH	<1	<1
3	Pt@Al_2_O_3_-400H	MeOH	20	19
4	Pt@_pr_CeO_2_-400H	MeOH	>99	>99
5	Pt@_pr_CeO_2_-400H	H_2_O	<1	<1
6	Pt@_pr_CeO_2_-400H	l-PrOH	91	90
7	Pt@_pr_CeO_2_-400H	THF	54	52
8	Pt@_pr_CeO_2_-400H	MeCN	20	18
9	Pt@_pr_CeO_2_-300H	MeOH	7	6
10	Pt@_pr_CeO_2_-500H	MeOH	56	55
11	Pt@_pr_CeO_2_-600H	MeOH	20	19
12	_pr_CeO_2_-400H	MeOH	<1	<1
13	Pt@C	MeOH	97	97

a Reaction conditions: 5 mg catalysts, 69 mg *p*-nitroaniline, 2 mL solvent, 35 °C, H_2_ balloon, 300 r.p.m. The Pt loading was around 0.5 wt%, determined by ICP (Table S1).

Furthermore, the reduction temperature during catalyst preparation (denoted by –TH, where *T* is temperature in °C) critically determines the final catalytic activity by modulating metal particle size, oxidation state, and the metal-support interface. A distinct volcanic trend was observed (Table 1, entries 4, 9–11). A low reduction temperature of 300 °C (Table 1, entry 9) resulted in poor activity (6% yield), possibly due to insufficient e formation of reactional favourable species. The activity peaked at a reduction temperature of 400 °C (Table 1, entry 4, >99% yield). However, further increasing the reduction temperature to 500 °C and 600 °C led to a progressive decline in yield to 55% and 19%, respectively (Table 1, entries 10–11). This decrease is commonly attributed to the sintering of Pt nanoparticles and the over-reduction or structural alteration of the _pr_CeO_2_ support, which may diminish the density of catalytically crucial interfacial sites and oxygen vacancies. These results underscore 400 °C as the optimal pre-treatment condition for achieving the highest density of active sites. The _pr_CeO_2_ alone is inactive for the model reaction, and commercial Pt@C gave 97% yield under the same conditions (Table 1, entries 12–13).

### Structural and morphological evolution of catalysts

**Fig. 1:**
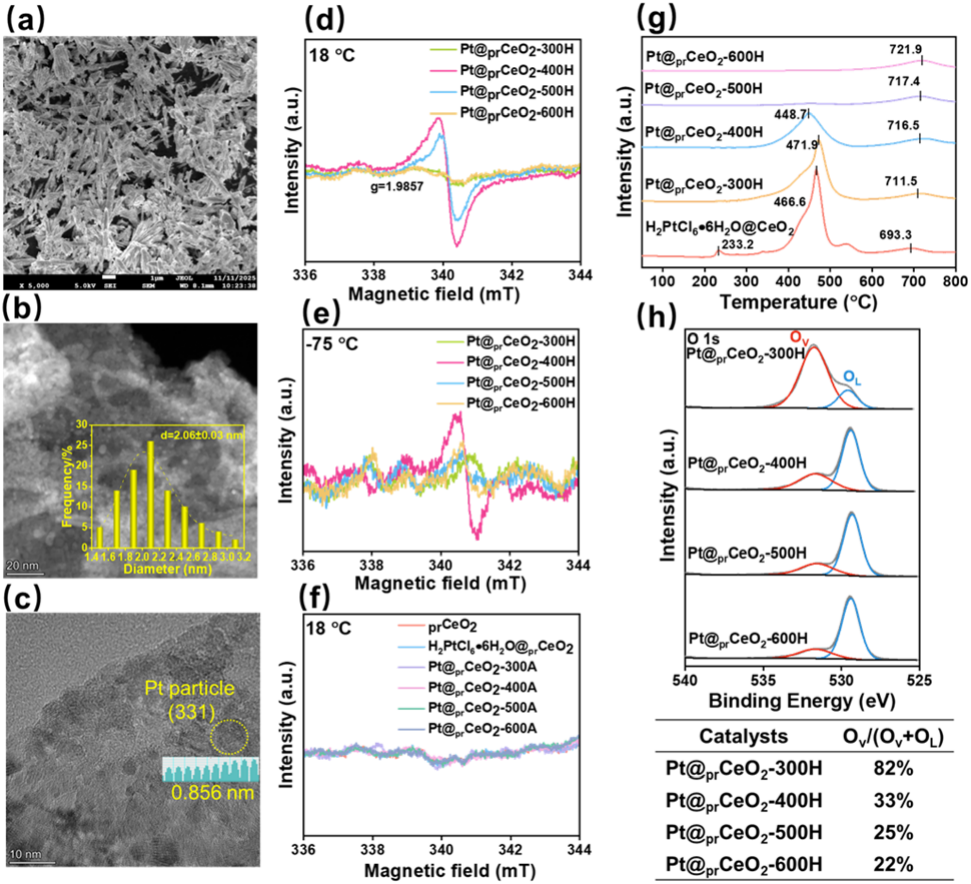
Characterization of original support, precursor and catalysts. (a) SEM, (b) and (c) TEM images of Pt@_pr_CeO_2_-400H; (d–f) EPR spectra, measured at 18 °C and −75 °C, of _pr_CeO_2_ support, H_2_PtCl_6_·6H_2_O@_pr_CeO_2_ precursor and catalysts; (g) H_2_-TPR spectra of H_2_PtCl_6_·6H_2_O@_pr_CeO_2_ precursor and catalysts; (h) O 1s XPS spectra of catalysts.

To systematically investigate the origin of the distinct catalytic activities, a series of characterization techniques were employed. The XRD analysis underscores a critical role of atmosphere at 300 °C (Fig. S1a and b). The pattern for Pt@_pr_CeO_2_-300H remains virtually identical to the initial _pr_CeO_2_, whereas the sample treated in air at the same temperature (Pt@_pr_CeO_2_-300A) exhibits a pattern consistent with crystalline CeO_2_.^16–18^ This demonstrates that oxidative conditions readily convert the precursor at 300 °C. However, a shift occurs at 400 °C and above: diffraction patterns for all catalysts—whether reduced in H_2_ or calcined in air—match that of CeO_2_, indicating that the structural transformation is governed primarily by temperature in this regime, with minimal atmospheric influence. Notably, the absence of Pt-related peaks confirms the high dispersion of Pt across all samples. As presented in Fig. 1a and S2, all Pt@_pr_CeO_2_-TH catalysts exhibit irregular rod-like structures. TEM images leveraging the contrast difference between Pt and the support clearly reveal the presence of uniformly distributed Pt nanoparticles on the support surface (Fig. 1b, c and S2). Statistical analysis of particle size distribution (Fig. 1b and S2) demonstrates that the average Pt particle size increases linearly from 1.9 nm to 2.9 nm as the reduction temperature rises from 300 °C to 600 °C. Meanwhile, the Pt particles with (331) plane was clearly observed on Pt@_pr_CeO_2_-400H (Fig. 1c).

### Electronic properties and surface defects

Given that the catalytic activity exhibits a volcanic trend with the reduction temperature, the electronic state of the catalysts was proposed as a key differentiating factor. Intriguingly, the intensity of the EPR signal measured at 18 °C (Fig. 1d) correlates positively with the catalytic activity: Pt@prCeO_2_-400H, the most active catalyst, shows the strongest signal; Pt@prCeO_2_-500H displays a slightly weaker signal; while the signals for the 300 °C and 600 °C samples are the weakest. The measurement temperature was further lowered to −75 °C (Fig. 1e), similar trend was observed too. Notably, Pt@_pr_CeO_2_-400H maintained the highest signal intensity under variable temperatures. This leads us to speculate that at the reaction temperature (35 °C), the abundant unpaired electrons on the 400 °C catalyst are likely involved in the activation of reactants, thereby contributing to its superior activity. The observed *g*-value of 1.9857 for our sample deviates from the typical range for Ce^3+^ (1.9654–1.9667), thereby ruling out any contribution from Ce^3+^ species.^19^ Furthermore, variable-temperature EPR analysis revealed distinct magnetic behaviors. For Pt@_pr_CeO_2_-400H and Pt@_pr_CeO_2_-500H, the signal intensity remained similar at 18 °C and −75 °C, indicating Pauli paramagnetism characteristic of conduction electrons. In contrast, Pt@_pr_CeO_2_-300H and Pt@_pr_CeO_2_-600H exhibited a significant increase in EPR signal intensity upon cooling from 18 °C to −75 °C, a behavior consistent with Curie paramagnetism arising from localized unpaired electrons. It is worth noting that catalysts treated in air atmosphere showed negligible EPR signals, indicating that the formation of these paramagnetic sites requires a reductive environment.

Hence, H_2_-TPR was conducted to research their reducibility (Fig. 1g). The catalysts reduced at 500 °C and 600 °C show almost no reduction peaks in the 100–600 °C range, implying that most reducible surface species were already consumed during the high-temperature H_2_ pretreatment. In contrast, the precursor (H_2_PtCl_6_·6H_2_O@_pr_CeO_2_) and the catalysts reduced at 300 °C and 400 °C exhibit prominent reduction peaks between 350 °C and 500 °C, indicating the presence of residual reducible oxide species. One might infer that the Pt@_pr_CeO_2_-500H and Pt@_pr_CeO_2_-600H catalysts should possess more oxygen vacancies due to more thorough reduction. However, XPS analysis of the O 1s region (Fig. 1h) reveals an opposite trend: the proportion of oxygen vacancies (*O*_v_) is highest (82.8%) for Pt@_pr_CeO_2_-300H and monotonically decreases to 21.8% for Pt@_pr_CeO_2_-600H (Table S2). Consistently, the relative concentration of Ce^3+^ ions, derived from Ce 3d spectra (Fig. S3, and Table S2), decreases from 42% to 19% as the reduction temperature increases from 300 °C to 600 °C. This suggests that while high-temperature reduction removes more reducible oxygen, it may also promote structural ordering or sintering, leading to a decrease in the concentration of certain types of oxygen vacancies detectable by XPS. The fraction of metallic Pt^0^ increases linearly from 28.9% to 68.3%, while that of Pt^2+^ decreases from 59.3% to 22.4% across the 300–600 °C series. This confirms that higher reduction temperatures favor the reduction of Pt species to the metallic state. Considering that the reduction of typical Pt oxides usually occurs around 200 °C, the H_2_-TPR peaks in the 350–550 °C range for the Pt@_pr_CeO_2_-300H and Pt@_pr_CeO_2_-400H catalysts are thus attributed to the reduction of strongly interacting Pt species, which indicating that some Pt species closely coordinated by the neighbouring atoms are located on Pt@_pr_CeO_2_-300H and Pt@_pr_CeO_2_-400H.

### Proposed origin of paramagnetic sites and catalytic role

**Fig. 2:**
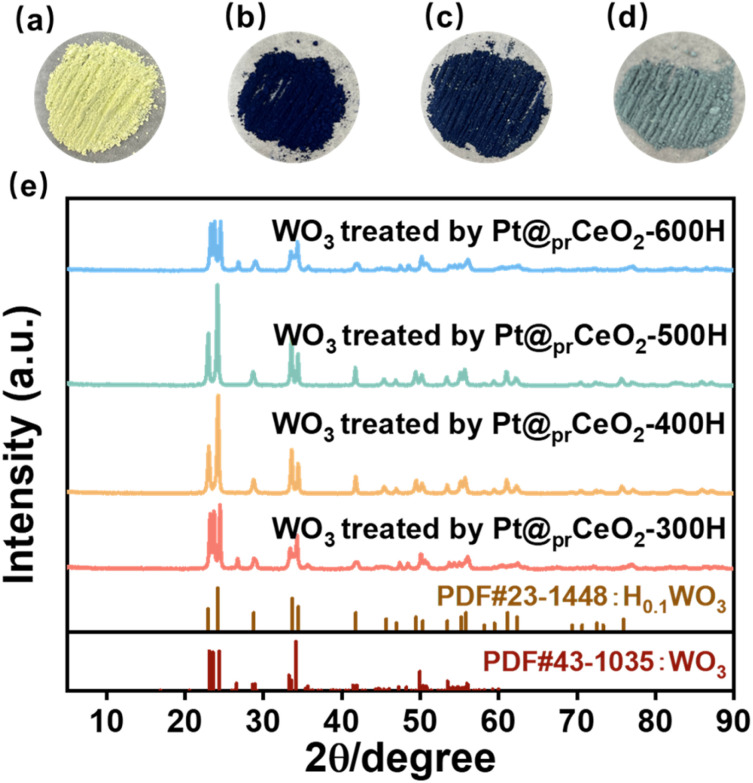
The hydrogen spillover experiments. (a) Pt@_pr_CeO_2_-300H, (b) Pt@_pr_CeO_2_-400H, (c) Pt@_pr_CeO_2_-500H, (d) Pt@_pr_CeO_2_-600H. (e) XRD patterns of fresh WO_3_ treated by various catalysts. Reaction conditions: 5 mg catalysts, 500 mg WO_3_, 35 °C, H_2_ balloon, 300 r.p.m, 1 h.

Since Pt^0^, Pt^2+^, and Pt^4+^ species are typically diamagnetic, the observed paramagnetism is unlikely to originate from isolated Pt species. Instead, we propose that the unpaired spins reside at the Pt-support interface. The removal of a neutral oxygen atom from CeO_2_ leaves behind two electrons. To maintain charge neutrality, adjacent Ce^4+^ ions can capture these electrons, reducing to Ce^3+^. Upon Pt loading, electron transfer occurs at the metal–support interface, potentially altering the electronic population of these vacancies.^20–22^ However, we only see minor differences of Pt 4f binding energy among these samples. Although the precise reason why the Pt@_pr_CeO_2_-400H catalyst hosts the maximum number of unpaired electrons remains elusive with current characterization, we hypothesize that the H_2_ treatment at this specific intermediate temperature creates an optimal interfacial microenvironment that maximizes the generation of paramagnetic sites. Temperatures that are too low may be insufficient for effective interface formation, while temperatures that are too high may lead to excessive reduction of Pt, particle growth, or vacancy annihilation, both being detrimental to the generation of these active paramagnetic sites. We postulate that these unpaired electrons play a crucial role in facilitating hydrogen spillover through the electron interactions. This hypothesis is further supported by subsequent hydrogen spillover experiments, which evaluated the spillover differences among these four samples (Fig. 2).

To demonstrate the paramagnetic active sites effect in the hydrogen spillover, WO_3_ was employed as an indicator mixed with the catalysts, and H_2_ reduction was implemented. This is because the hydrogen species that spill would migrate and interact with yellow WO_3_ powder, resulting in the formation of a dark blue H_*x*_WO_3_.^23,24^ As illustrated in Fig. 2a–d, the sample treated by Pt@_pr_CeO_2_-400H and Pt@_pr_CeO_2_-500H exhibited a dark blue color, while Pt@_pr_CeO_2_-300H and Pt@_pr_CeO_2_-600H display yellow and gray green color, respectively. The color variation, resulted from the different hydrogen spillover capability, was demonstrated by the XRD characterization of WO_3_ treated by various catalysts under typical conditions, as H_0.1_WO_3_ species was observed in Pt@_pr_CeO_2_-400H and Pt@_pr_CeO_2_-500H (Fig. 2e). This finding is consistent with the EPR conclusion, demonstrating the vital role of paramagnetic active sites in spillover the hydrogen species.^25^

### Substrate scope and recyclability

Further, we explored the applicability of the process for hydrogenation of different nitro compounds (Table 2). The aromatic nitro compounds bearing amides, amine, ketone and chloral functional groups could be well tolerated by this catalytic system. Probably due to the negative effect of pyridine, the hydrogenation of 3-nitropyridin-2-amine only gave 48% yield.

**Table 2:** The hydrogenation of various nitro compounds^a^

Entry	Substrate	Product	Yield, %
1	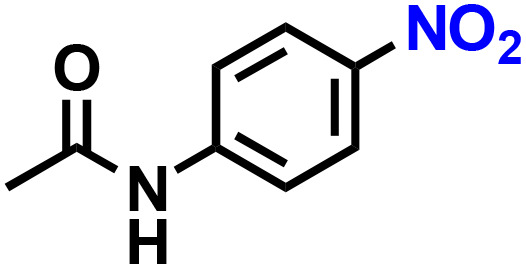	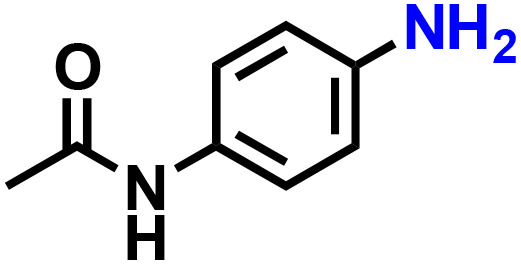	>99
2	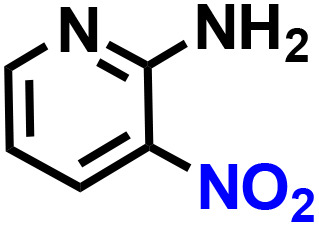	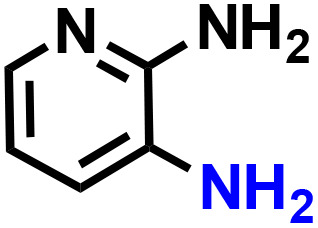	48
3	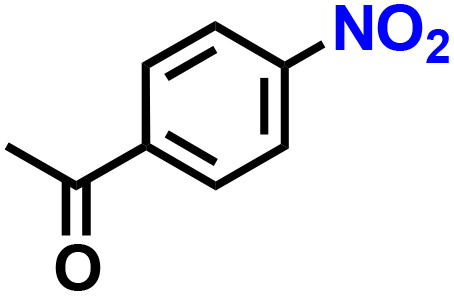	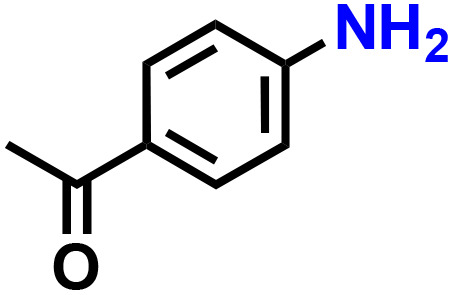	>99
4	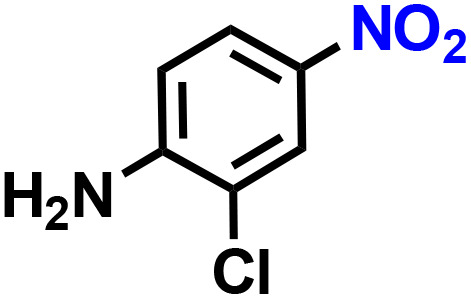	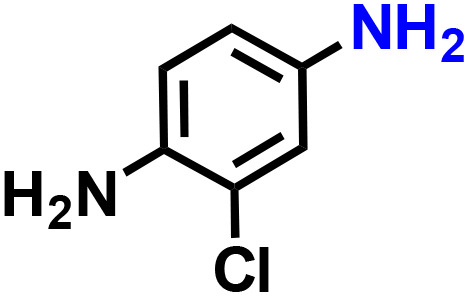	>99
5	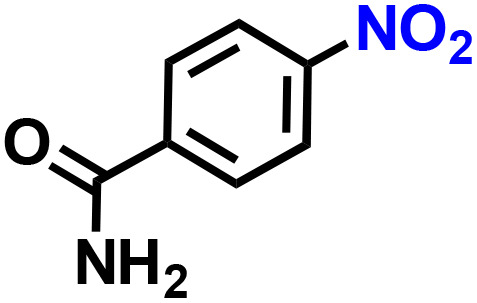	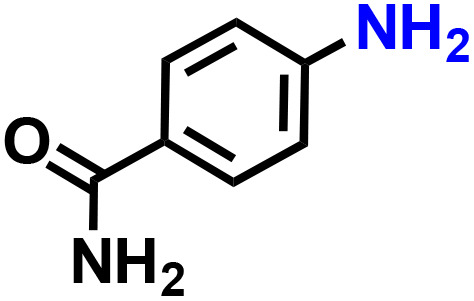	>99

a Reaction conditions: 5 mg catalysts, 69 mg *p*-nitroaniline, 2 mL MeOH, 35 °C, H_2_ balloon, 300 r.p.m, 10 h. Yields were determined by GC-MS.

**Fig. 3:**
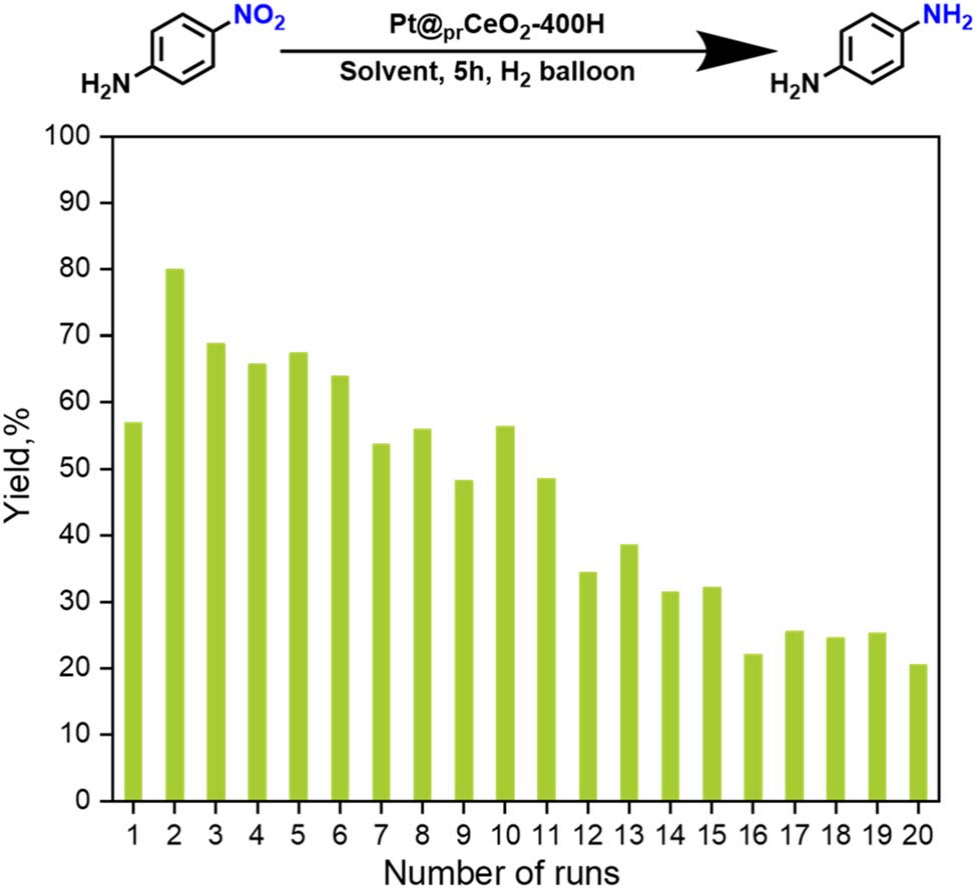
Catalysts recycling for the hydrogenation of *p*-nitroaniline. Reaction conditions: 5 h, methanol solvent, hydrogen balloon. The mass ratio of catalysts/*p*-nitroaniline in each run is 7.2%, and the loading of catalysts and *p*-nitroaniline for the first run was 0.5 g and 6.9 g, respectively.

Finally, recycling and reusability of the proposed catalysts were tested using the model reaction and it was found that it still retains 50% of its original performance after 20 cycles (Fig. 3). The loading of catalysts and *p*-nitroaniline gradually decreased because of the catalysts loss (Table S3). The recycled catalysts are further subject to EPR, TEM, XRD, and XPS characterizations to understand the behind reasons. No significant differences were observed from EPR, XRD, and XPS. Characterization of the recycled materials revealed that the average particle size increasedlinked marginally from 2.0 nm to 2.5 nm after 20 cycles. Furthermore, thermogravimetric analysis (TGA) of the recycled samples indicated that approximately 2.5 wt% of the reaction mixture remained adsorbed on the catalyst surface and could not be removed by simple washing with methanol. This residual organic matter is believed to block the catalyst's pores, thereby leading to its deactivation.

## Conclusions

In summary, we developed a Pt@_pr_CeO_2_-400H catalyst that achieves quantitative hydrogenation of *p*-nitroaniline under green conditions (35 °C, balloon H_2_). Its exceptional performance is attributed to paramagnetic active sites at the Pt–_pr_CeO_2_ interface—likely defect-based sites with unpaired electrons that promote hydrogen spillover through electron interactions. The generation of these paramagnetic active sites dependent on the H_2_ gas, which probably rearrange the microenvironment of Pt-support interface upon heating. This work thus provides a new strategy toward green hydrogenation processes.

## Author contributions

H. Y. performed the catalysts preparation, evaluation and substrate scope part. Y. L. conducted the characterization part and draw the images. X. Y. conducted the recycling experiments. J. G. supervised the project, modified the images and prepare the manuscript.

## Conflicts of interest

There are no conflicts to declare.

## Supplementary Material

RA-016-D6RA00621C-s001

## Data Availability

The data supporting this article have been included as part of the supplementary information (SI). Supplementary information: the followsing items are included in SI file. S1: Materials and methods; S2: Procedure for catalyst preparation; S3: Procedure for catalyst evaluation; S4: Procedure for catalyst recycling; S5: Supplementary Tables; S6: Supplementary Figures. See DOI: https://doi.org/10.1039/d6ra00621c.
