# Supplementary material for: Paramagnetic active sites boosted hydrogenation of p-nitroaniline over prCeO2 supported Pt catalysts
Source: RSC Adv. 2026 Mar 16;16(16):14246–50. doi: 10.1039/d6ra00621c (PMC12991058; doi:10.1039/d6ra00621c)
Supplement: RA-016-D6RA00621C-s001 [file RA-016-D6RA00621C-s001.pdf]

Supplementary Information (SI) for RSC Advances.
This journal is © The Royal Society of Chemistry 2026

Paramagnetic active sites boosted hydrogenation of p-nitroaniline over $_{pr}CeO_2$ supported Pt catalysts

Table of contents

S1. Materials and methods

S2. Procedure for catalyst preparation

S3. Procedure for catalyst evaluation

S4. Procedure for catalyst recycling

S5. Supplementary Tables

S6. Supplementary Figures

S1. Materials and methods

The nitro compounds, methanol and H_2PtCl_6•$6H_2O$ were obtained commercially from various chemical companies.

XRD powder patterns were recorded on a Stoe STADI P diffractometer, equipped with a linear Position Sensitive Detector (PSD) using Cu Kα radiation (λ = 1.5406 Å).

XPS measurements were performed in VG ESCALAB220iXL with monochromate AlKα radiation (E = 1486.6 eV).

TEM measurements were performed on a JEM-2100 operating at an accelerated voltage of 200 kV. The sample was ultrasonicated in ethanol solution, and a drop was deposited on a copper grid covered with a holey carbon membrane for observation.

The surface area and porosity were carried out by the N_2 adsorption isotherm using the Brunauer-Emmett-Teller (BET) method on an ASAP 2020 Micromeritics instrument. Before analysis, all samples were degassed at 200 °C for 6 h to desorb moisture and impurities from their surfaces. The pore size distributions were calculated using the Barrett-Joyner-Halenda (BJH) model from the desorption branch.

The EPR was measured using MS 5000X, Magnettech, 9.2 - 9.6 GHz, produced by Germany.

The GC-MS was measured using GCMS-QP2020NX, Shimadzu.

S2. Procedure for catalyst preparation

All catalysts mentioned in this work were prepared via the same procedure. A typical procedure for the preparation of optimal catalyst (Pt@$_{pr}$$CeO_2$-400H) is as follows: In a round bottomed flask, 0.1916 g H_2PtCl_6•$6H_2O$ solution are stirred in water (25 mL) for 5 minutes at room temperature. Then, the CeO_2 support prepared using a precious method was synthesised, and 1g of CeO_2 was added. Then, the mixture was stirred for another 12 h at room temperature. After that, menthol was removed by rotary evaporation at 55 °C/350 mPa, the material obtained was dried at 80 °C in oven for 12 h. Finally, the resulting solid material was grinded to fine powder and pyrolyzed using tube furnace.

S3. Procedure for catalyst evaluation

A 4 mL dried glass vial was charged with magnetic stirring bar, 0.5 mmol substrate nitro compounds. Then, 2 mL MeOH were added followed by the addition of catalyst (5 mg). Then, the vial was fitted with septum, cap and needle and placed in into a 500 ml autoclave (8 vials at a time). The autoclave was flushed with H_2 gas twice and the H_2 gas supplied using a balloon. Then, the autoclave was placed into an block and the reactions were stirred for required time at 35 °C. After the completion of reaction, the autoclave was cooled to room temperature. The remaining H_2 in the autoclave was released and the reaction vials were taken out from the autoclave. The solid catalyst was filtered off and washed thoroughly with MeOH. The resulting reaction products were analysed by GC.

S4. Procedure for catalyst recycling

The recycling experiment was conducted using the similar procedure with some minor changes. A 500 mL dried glass vial was charged with magnetic stirring bar, 6.9 g nitro compounds. Then, 2 mL MeOH were added followed by the addition of catalyst (500 mg). Then, the vial was directly placed in into a 500 ml autoclave. The autoclave was flushed with H_2 gas twice and the H_2 gas supplied using a balloon. Then, the autoclave was placed into an block and the reactions were stirred for 5 h at 35 °C. After the completion of reaction, the autoclave was cooled to room temperature. The remaining H_2 in the autoclave was released and the reaction vials were taken out from the autoclave. The solid catalyst was filtered off and washed thoroughly with MeOH. The resulting reaction products were analysed by GC.

S5. Supplementary Tables

Table S1. The content of Pt determined by ICP

Catalyst	Wt%
Pt@CeO_2-300H	0.4622
Pt@CeO_2-400H	0.4962
Pt@CeO_2-500H	0.4988
Pt@CeO_2-600H	0.5007

Table S2. The content of components determined by XPS

Entry	Catalysts	Components			
		$Ce^{3+}/(Ce^{3+}+Ce^{4+})$	$O_v/(O_{v+}O_{L)}$	$Pt^{0+}/(Pt^{4+})$	$Pt^{0+}+Pt^{2+}+$
1	Pt@$_{pr}CeO_2$-300H	42	82	24	
2	Pt@$_{pr}CeO_2$-400H	20	33	53	
3	Pt@$_{pr}CeO_2$-500H	19	25	64	
4	Pt@$_{pr}CeO_2$-600H	19	22	88	

Table S3. The remaining catalysts mass and p-nitroaniline loading in each recycling experiment.

Number of recycling experiment	Remaining mass of catalysts, g	Loading of p-nitroaniline, g
1	0.5001	6.9000
2	0.4634	6.3739
3	0.4478	6.1595
4	0.4337	5.9704
5	0.4205	5.7881
6	0.3973	5.4702
7	0.3809	5.2445
8	0.3704	5.0990
9	0.3488	4.8016
10	0.3425	4.7150
11	0.3014	4.1580
12	0.2998	4.1250

13	0.2985	4.1111
14	0.2630	3.6191
15	0.2522	3.4728
16	0.2440	3.3680
17	0.2279	3.1359
18	0.2080	2.8663
19	0.1998	2.7521
20	0.1839	2.5301

Notes: The ratio of catalysts and p-nitroaniline was kept at 0.5:6.9.

Table S4. Comparison of reaction conditions of Pt based catalysts for hydrogenation of nitro compounds.

Entry	Catalyst	Time, h	T, °C	Pt loading (wt%)	Mass ratio (catalyst/ substrate)	Solvent	H_2 pressure	Yield. %	Ref
1	Pt/MgO	2	30	0.3	0.4	Toluene	5 bar	88.5	1
2	Pt/Al_2O_3	2	30	0.5	0.4	Toluene	5 bar	74.3	1
3	Pt/TiO_2	2	30	0.2	0.4	Toluene	5 bar	45.5	1
4	SAC-Pt/C	0.83	40	0.3	0.65	Ethanol	1	96	2
5	Pt/TiO_2/ RGO	0.13	60	2.1	0.01	Solvent free	40	100	3
6	Pt/$_{pr}CeO_2$	10	35	0.5	0.007	Methanol	H_2 balloon	99	This work

Ref:

1. P. Jing et al. Chinese Journal of Catalysis 40 (2019) 214–222

2. X.Yan et al. Carbon143(2019)378e384

3. C.H. Campos et al. CatalysisToday394-396(2022)510–523

S6. Supplementary figures

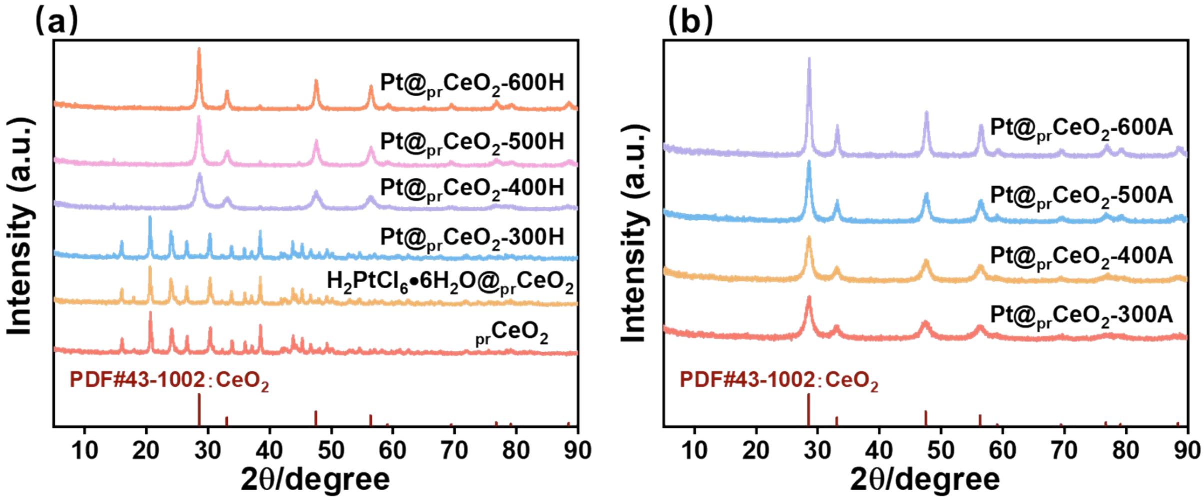

Figure S1. XRD patterns of (a) $_{pr}CeO_2$ support, $H_2PtCl_6•6H_2O@_{pr}CeO_2$ precursor and catalysts prepared using H_2; (b) catalysts prepared using air.

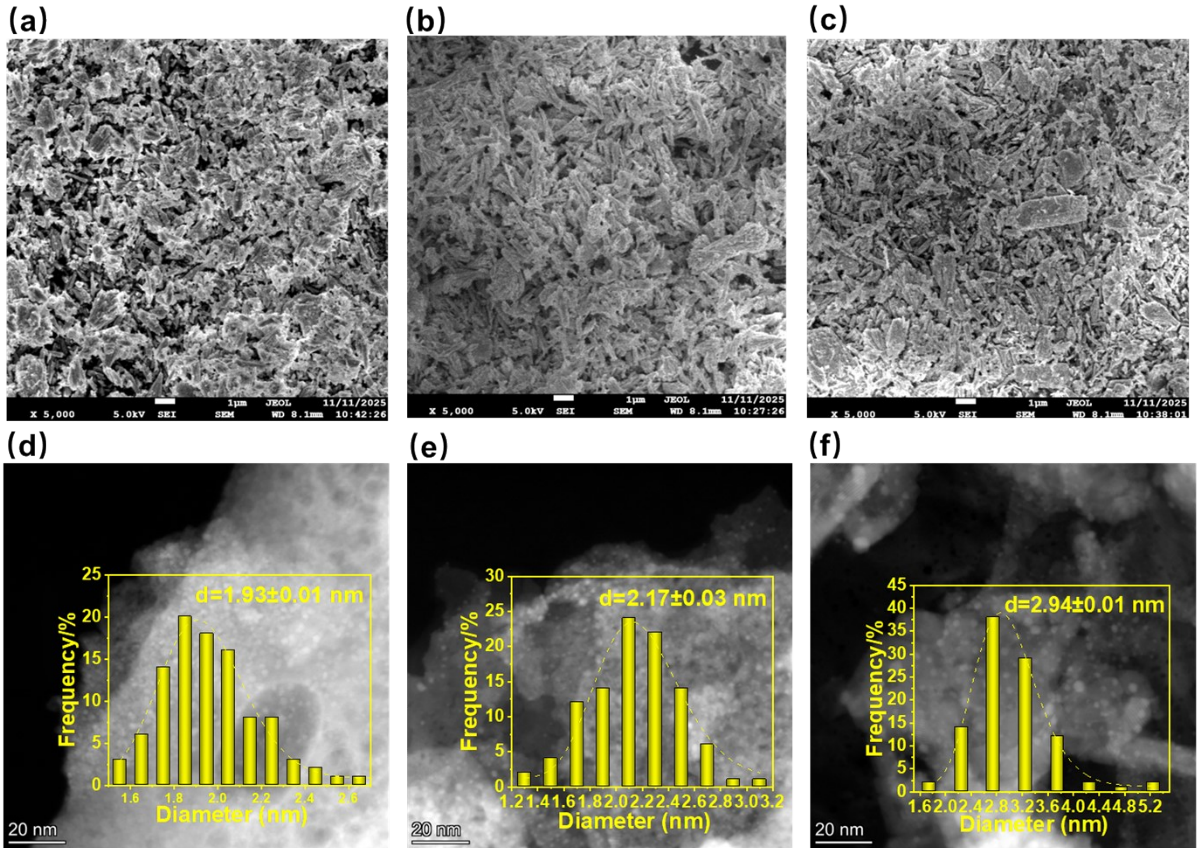

Figure S2. The SEM and TEM images of Pt@$_{pr}CeO_2$-300H, Pt@$_{pr}CeO_2$-500H and Pt@$_{pr}CeO_2$-600H.

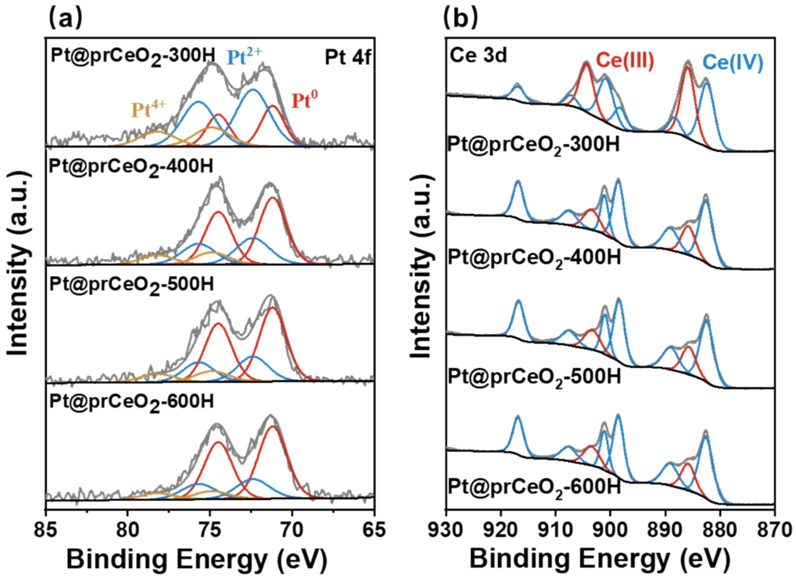

Figure S3. The (a) Pt 4f and (b) Ce 3d XPS spectra of catalysts prepared using H_2.

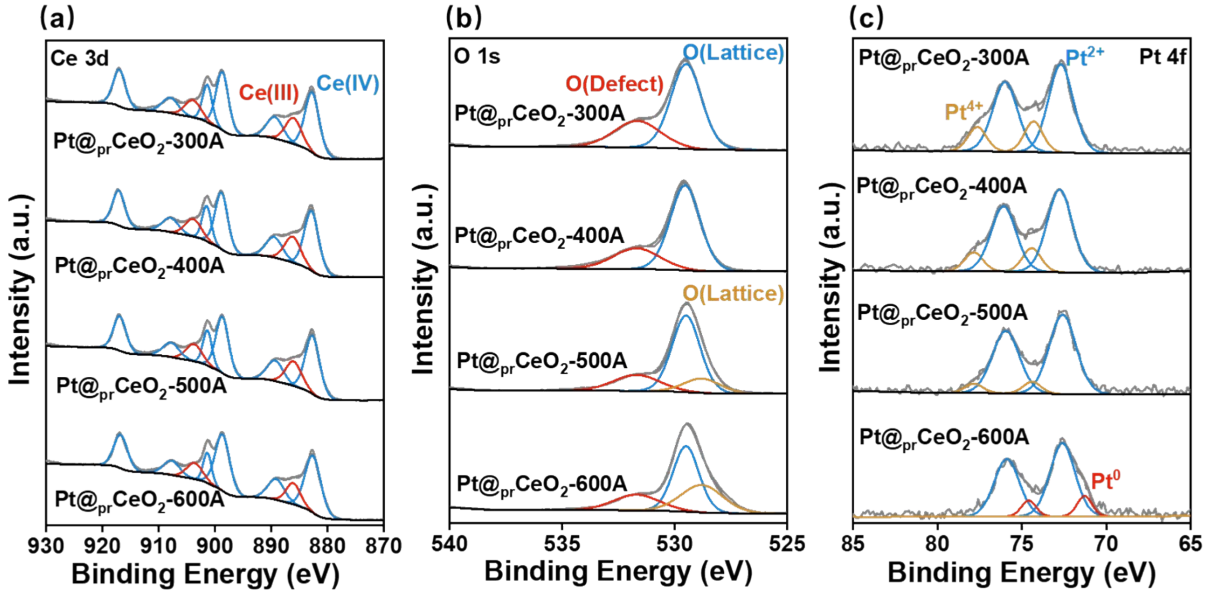

Figure S4. The (a) Ce 3d, (b) O 1S and (c) Pt 4f spectra of catalysts prepared using air.

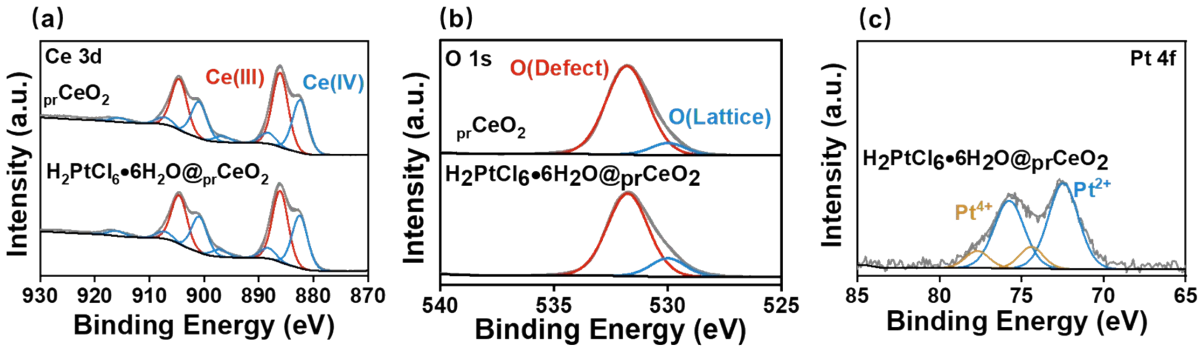

Figure S5. The (a) Ce 3d, (b) O 1S and (c) Pt 4f spectra of $prCeO_2$ support and H_2PtCl_6•$6H_2O$@$_{pr}CeO_2$ precursor.

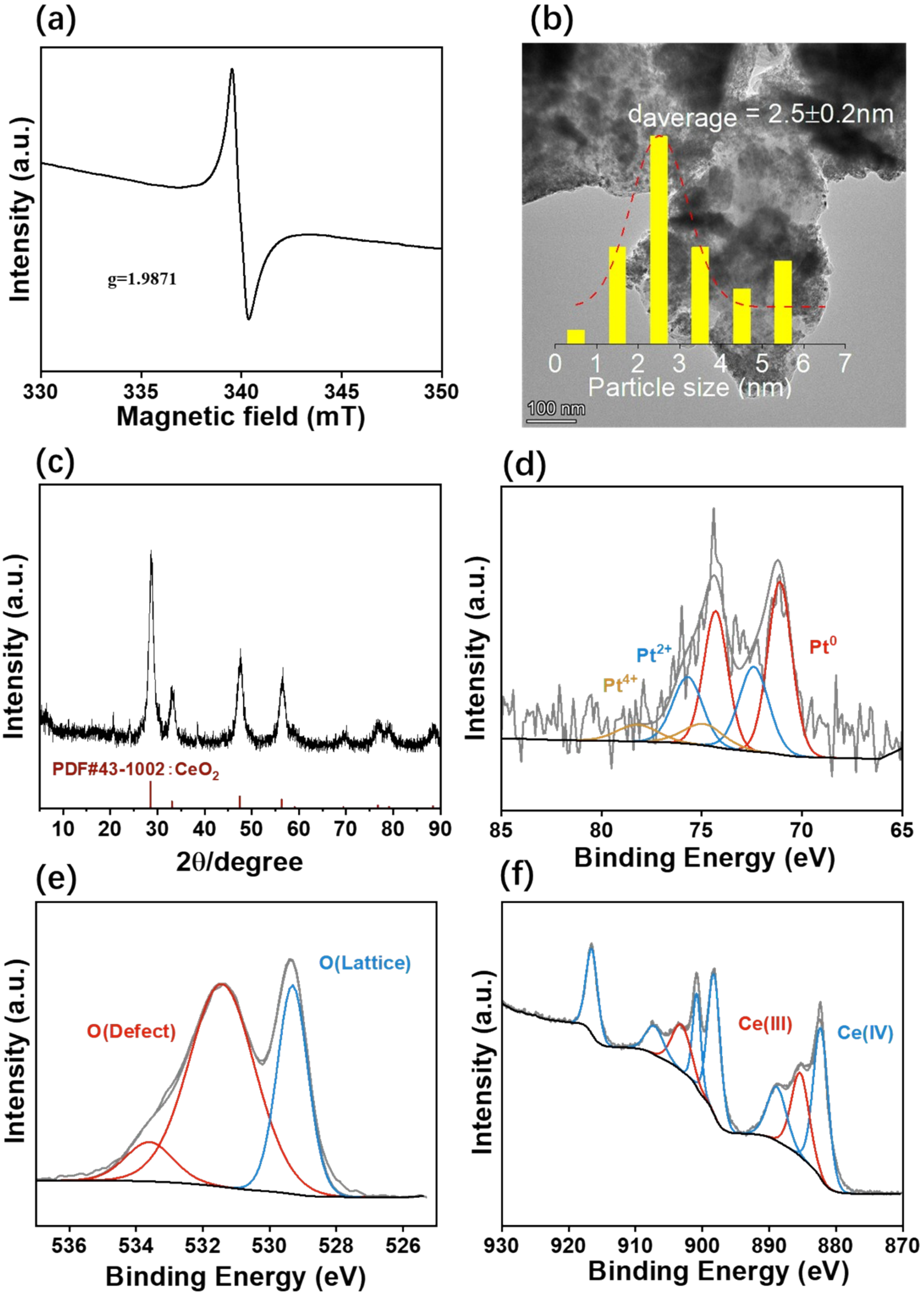

Figure S6. The characterizations of reused Pt@$_{pr}$CeO$_2$-400H catalysts. (a) EPR, (b) TEM, (c) XRD, (d) Pt 4f XPS spectra, (e) O 1s XPS spectra, (f) Ce 3d XPS spectra.
